# Use of thrombopoietin receptor agonists in adults with immune thrombocytopenia: a systematic review and Central European expert consensus

**DOI:** 10.1007/s00277-023-05114-8

**Published:** 2023-02-24

**Authors:** Dražen Pulanić, Angelika Bátorová, Imre Bodó, Libor Červinek, Ioana Ionita, Toshko Lissitchkov, Anahit Melikyan, Maria Podolak-Dawidziak

**Affiliations:** 1 Division of Hematology, Department of Internal Medicine, University Hospital Center Zagreb, University of Zagreb, School of Medicine, Kispaticeva 12, 10 000 Zagreb, Croatia; 2 Department of Hematology and Transfusion Medicine, National Hemophilia Center, Faculty of Medicine of Comenius University and University Hospital Bratislava, Bratislava, Slovakia; 3 3rd Department of Internal Medicine, Semmelweis University, Budapest, Hungary; 4 Faculty Hospital Brno, Department of Internal Medicine - Hematology and Oncology, Masaryk University, Brno, Czech Republic; 5 Department of Internal Medicine, Victor Babes University of Medicine and Pharmacy, HematologyTimisoara, Romania; 6 Specialized Hospital for Active Treatment of Hematological Diseases, Sofia, Bulgaria; 7 Department of Standardization of Treatment Methods, National Research Center for Hematology Russian Federation, Moscow, Russia; 8 Department of Hematology, Blood Neoplasms and Bone Marrow Transplantation, Wroclaw Medical University, Wroclaw, Poland

**Keywords:** Immune thrombocytopenia, Thrombopoietin receptor agonists, Systematic review, Consensus, Eltrombopag, Romiplostim, Avatrombopag

## Abstract

There are currently three thrombopoietin receptor agonists (TPO-RAs) approved in Europe for treating patients with immune thrombocytopenia (ITP): romiplostim (Nplate®), eltrombopag (Revolade®), and avatrombopag (Doptelet®). However, comparative clinical data between these TPO-RAs are limited. Therefore, the purpose of this study was to perform a literature review and seek expert opinion on the relevance and strength of the evidence concerning the use of TPO-RAs in adults with ITP. A systematic search was conducted in PubMed and Embase within the last 10 years and until June 20, 2022. A total of 478 unique articles were retrieved and reviewed for relevance. The expert consensus panel comprised ITP senior hematologists from eight countries across Central Europe. The modified Delphi method, consisting of two survey rounds, a teleconference and email correspondence, was used to reach consensus. Forty articles met the relevancy criteria and are included as supporting evidence, including five meta-analyses analyzing all three European-licensed TPO-RAs and comprising a total of 31 unique randomized controlled trials (RCTs). Consensus was reached on seven statements for the second-line use of TPO-RAs in the management of adult ITP patients. In addition, the expert panel discussed TPO-RA treatment in chronic ITP patients with mild/moderate COVID-19 and ITP patients in the first-line setting but failed to reach consensus. This work will facilitate informed decision-making for healthcare providers treating adult ITP patients with TPO-RAs. However, further studies are needed on the use of TPO-RAs in the first-line setting and specific patient populations.

**Supplementary Information:**

The online version contains supplementary material available at 10.1007/s00277-023-05114-8.

## Introduction

Immune thrombocytopenia (ITP) is a rare, acquired autoimmune disorder resulting from the destruction of platelets in the reticuloendothelial system due to anti-platelet antibodies and other immune processes [1, 2]. The cause of this platelet-specific autoimmunity is complex and poorly understood, but recent evidence suggests that B- and T-cell dysregulation may play a central role [2]. ITP is estimated to have an annual incidence rate of about 3 per 100 000 persons [3–5]. However, the incidence has reportedly increased in recent years, especially among young women and older men [3, 5, 6]. Most adult cases (about 80%) are classified as primary ITP [7], with 20% of cases attributable to secondary causes such as medications or concurrent diseases (e.g., autoimmune conditions such as systemic lupus erythematosus or chronic infections) [5, 8].

The condition is characterized by isolated low platelet counts (less than 100 × 10^9^/L) due to increased turnover and inadequate production of platelets, leading to an increased risk of bleeding, the predominant symptom [9, 10]. The severity of bleeding varies for individual patients, from asymptomatic to intractable bleeding at presentation [11]. The treatment goal, therefore, is to increase and maintain a hemostatic platelet count above 30‒50 × 10^9^/L and is typically initiated in patients who have a platelet count less than 30 × 10^9^/L and bleeding diathesis [12, 13]. The clinical course in adults is described as three separate phases: (1) newly diagnosed phase (ITP less than 3 months), (2) persistent phase (ITP lasting between 3 and 12 months), and chronic phase (ITP lasting greater than 12 months) [12–14]. The standard first-line treatment for the majority of newly diagnosed adult ITP cases (approx. 80%) is corticosteroids (e.g., dexamethasone, prednisone, or methylprednisolone) administered ideally for no more than 6 weeks and supplemented as needed with intravenous immunoglobulin (IVIG) or anti‐D (available in some countries) [12, 13]. Note, many experts prefer pulse-dose dexamethasone to other steroids. Second-line treatments are required for patients who do not respond to first-line therapy (20% cases) or relapse when corticosteroids are reduced or stopped (70–90% cases) [15, 16]. Second-line pharmacological agents include thrombopoietin receptor agonists (TPO-RAs), rituximab, and other immune‐modulating medications such as fostamatinib or splenectomy [12, 13]. Three TPO-RAs are currently approved for use in Europe for adult patients with primary ITP who are refractory to first-line therapies: romiplostim (Nplate®), approved by the European Medicines Agency (EMA) in 2009 as a once-weekly subcutaneous injection [17]; eltrombopag (Revolade®)[18], a once-daily oral treatment, first approved by the EMA in 2010; and avatrombopag (Doptelet®), approved by the EMA in 2021 as a once-daily oral treatment for primary chronic ITP [19]. Notably, existing international and American Society of Hematology (ASH) ITP treatment guidelines from 2019 do not consider the most recently approved TPO-RA, avatrombopag [12, 13]. Despite having similar mechanisms of action, administration considerations and binding sites to TPO receptors may differ; however, no direct comparisons of TPO-RAs have been made in the clinical setting [20]. Robust clinical trials on the management of ITP, including when and how to safely taper or discontinue TPO-RAs, are also lacking [21].

To help address this gap, we conducted a systematic review on the use of TPO-RAs in adult ITP published in the last 10 years and since the approval of avatrombopag. We assessed the quality of evidence in the literature in order to develop expert consensus statements. In circumstances where clinical evidence is lacking, evidence-based data, together with expert opinion and clinical experience, can help guide busy clinicians in clinical decision-making to improve patient outcomes.

## Methods

### Data sources and search strategy

A systematic search was conducted in PubMed Central (PubMed®) and Excerpta Medica Database (Embase) to identify clinical studies evaluating the use of TPO-RAs in patients with ITP. The following combination of search terms was used: [“avatrombopag” OR “Doptelet” OR romiplostim” OR “Nplate” OR eltrombopag” OR “Revolade” OR “recombinant thrombopoietin” OR “recombinant human thrombopoietin” OR “thrombopoietin receptor agonist”] AND [“idiopathic thrombocytopaenic purpura”, “idiopathic thrombocytopenia” OR “'idiopathic thrombocytopaenia purpura” OR “idiopathic thrombocytopenia” OR “idiopathic thrombocytopenia purpura” OR “idiopathic thrombocytopenic purpura” OR “immune thrombocytopenia” OR “immune thrombocytopaenia” OR “purpura, thrombocytopenic, idiopathic” OR “thrombocytopaenia, chronic idiopathic” OR “thrombocytopaenia, idiopathic” OR “thrombocytopenia, chronic idiopathic” OR “'thrombocytopenia, idiopathic” OR “thrombocytopenic purpura, acute idiopathic” OR “thrombocytopenic purpura, chronic idiopathic”]. The search was limited to English language and full-text articles published within the last 10 years up to June 20, 2022, i.e., abstracts or articles published prior to 2012 were excluded.

### Study selection

Results were combined and exported to Endnote, where duplicates were removed. After removing duplicates, the search resulted in a total of 478 unique records that underwent manual title and/or abstract review by an independent reviewer (KB). Higher levels of evidence were prioritized, but lower-quality studies were also evaluated. Articles were excluded if they met one or more of the following exclusion criteria:

- Not specific to the management of ITP
- Not focused on the adult population
- Abstracts only; no full text
- Systematic reviews/meta-analyses that did not analyze all three EMA-approved TPO-RAs (romiplostim, eltrombopag, and avatrombopag)

### Levels of evidence

Individual clinical studies were evaluated in accordance with the Oxford Center for Evidence-Based Medicine 2011 Levels of Evidence (CEBM) [22]. An adapted version of the levels of evidence is shown in Table 1.

**Table 1:** Levels of evidence—adapted from Oxford Center for Evidence-Based Medicine (OCEBM) 2011 [22]

Level	Evidence (treatment benefits)
I	Systematic review or meta-analysis of RCTs, high-quality individual RCTs
II	Systematic review or meta-analysis of cohort studies, low-quality individual RCTs, prospective studies
III	Systematic review of case–control studies, retrospective cohort studies
IV	Case series
V	Expert opinion

Abbreviation: *RCTs* randomized controlled trials

### Grades of recommendation

The grades of recommendation are shown in Table 2. The authors assigned the grades of recommendation based on the strength of evidence and the results of the Delphi consensus. To gain the highest grade of recommendation (Grade A), consistent level 1 studies must be available.

**Table 2:** Grades of recommendation—adapted from Oxford Center for Evidence-Based Medicine (OCEBM) 2011 [22, 23]

Level	Evidence (treatment benefits)
A	Consistent level 1 studies
B	Consistent level 2 or 3 studies or extrapolations from level 1 studies
C	Level 4 studies or extrapolations from level 2 or 3 studies
D	Level 5 evidence or troublingly inconsistent or inconclusive studies of any level

### Consensus process

DP drafted the consensus statements following an initial discussion meeting with AB, II, and LC. The invited expert consensus panel comprised ITP senior hematologists from eight countries across Central Europe (Bulgaria, Croatia, Czech Republic, Hungary, Poland, Romania, Russia, and Slovakia). A modified Delphi method was used to collect the experts’ opinions on the consensus statements. Notably, the Delphi method is a validated consensus process commonly used when clinical evidence is limited [24]. During the first round, the expert panel reviewed and commented on the consensus statements using a 5-point scale: 1, strongly agree; 2, agree; 3, neither agree nor disagree; 4, disagree; and 5, strongly disagree. A score of 1–2 was determined as “Agreement.” Consensus for each statement was reached if at least six of the eight experts (> 75%) provided a score of 1–2. An independent reviewer (KB) collected and analyzed anonymized statement scores. Following the first-round review, the amalgamated scores were emailed to the expert panel members, which allowed the experts to rerate their scores in a second-round review. Finally, the expert panelists assigned the consensus statement grades based on the strength of evidence and consensus of the results of the Delphi rounds.

## Results

### Literature review

**Fig. 1:**
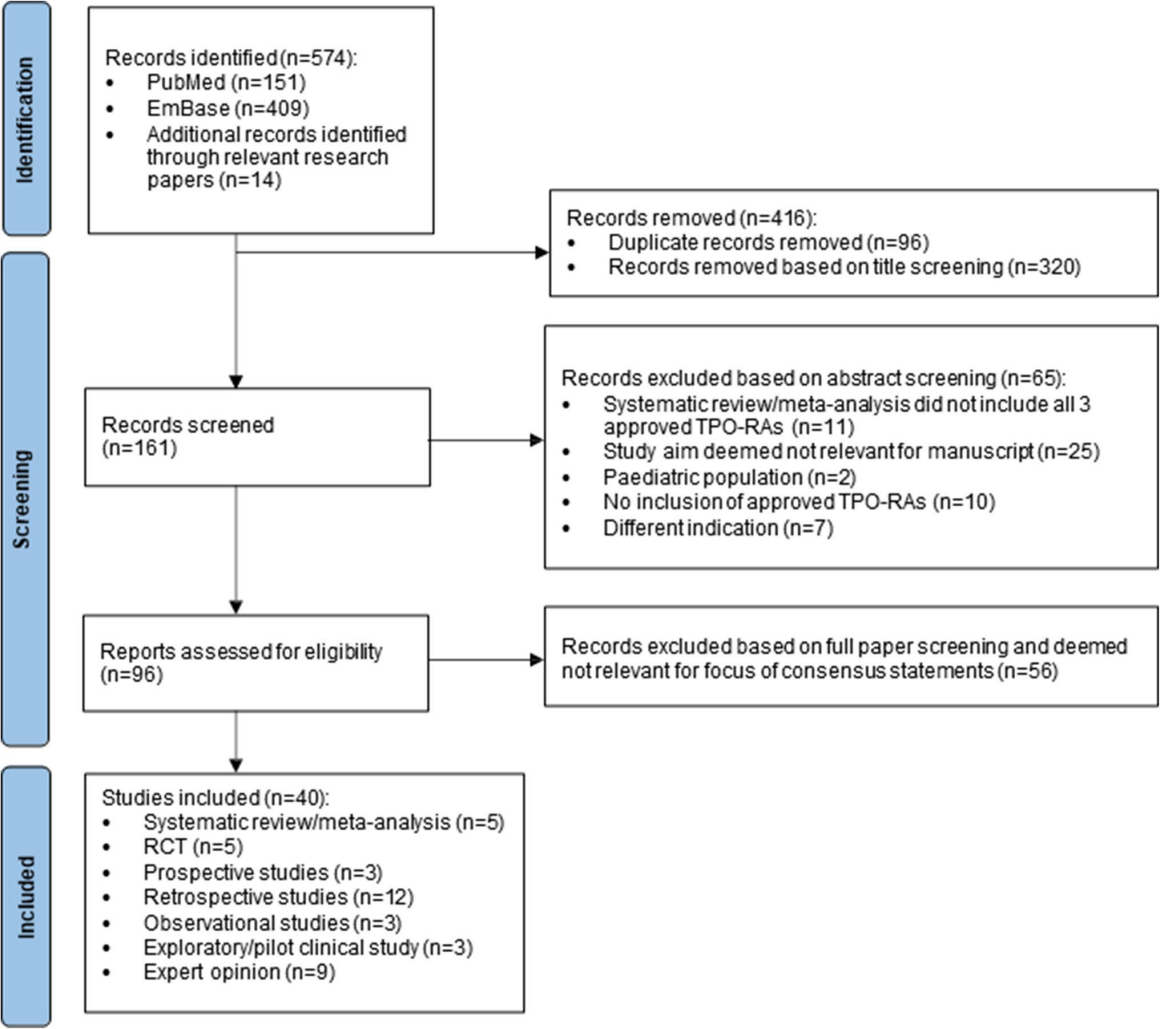
PRISMA flow diagram

Of the 161 records screened, 96 were reviewed in full for eligibility, with 40 final articles identified with relevant data (Fig. 1). The included studies’ characteristics and quality of evidence are shown in the Supplementary Material (Table S1).

## Consensus on the use of TPO-RAs in adults with ITP

### Preferred second-line treatment for chronic ITP

Current, major international and ASH ITP guidelines from 2019 recommend TPO-RAs eltrombopag and romiplostim, among other therapies, as second-line treatment following corticosteroids; notably, the most recently approved TPO-RA, avatrombopag, was not considered. In addition, comparative efficacy and tolerability studies are limited [12, 13]. Despite the lack of comparative studies, similar response rates have been reported for all three TPO-RAs in adults with ITP [25]. Five meta-analyses of randomized controlled trials (RCTs) comparing romiplostim, eltrombopag, and avatrombopag, as well as fostamatinib and rituximab, were retrieved from our literature search to support the use of all three TPO-RAs as preferred second-line agents [26–30]. The expert panel reached 100% consensus (strongly agree *n* = 8) for the use of TPO-RAs as the preferred second-line treatment for chronic ITP patients who are refractory to a previous treatment such as corticosteroids or immunoglobulins, as per the approved indication for romiplostim, eltrombopag, and avatrombopag [17–19].
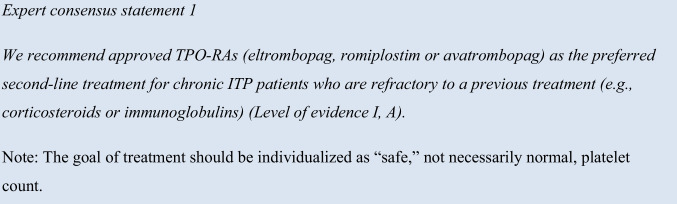


### Second-line TPO-RAs in patients with newly diagnosed and persistent ITP

There is limited clinical guidance on the timing of TPO-RA administration in the literature [31]. However, an increasing number of studies have been published providing evidence for the benefit of early TPO-RA therapy (less than 3 months) in the second-line setting. Five retrospective studies [32–36], two prospective studies [37, 38], one observational study [39], and five expert opinion reports [12, 13, 31, 40, 41] were retrieved from the literature search to support this consensus statement. Currently, the EMA does not set time restrictions for second-line administration of romiplostim or eltrombopag in adult primary ITP [17, 18]. The expert panel reached 100% consensus (agree *n* = 8) on the benefit of TPO-RAs as second-line treatment for ITP patients with newly diagnosed or persistent ITP. An individualized approach in the second-line setting for newly diagnosed ITP patients, e.g., some patients may have a contraindication to rituximab, was highlighted as an important consideration by all experts. The issue of whether TPO-RAs should be used before 3-month duration of ITP was also discussed. However, the experts did not concur when asked whether “TPO-RAs should not be used before 3 months of duration of ITP because they are not recommended in some current guidelines, namely ASH 2019 [13, 40] and international consensus report (ICR) 2019 [12], for the treatment of newly diagnosed ITP” (two experts agreed, four experts disagreed, while two experts neither agreed nor disagreed with this statement).
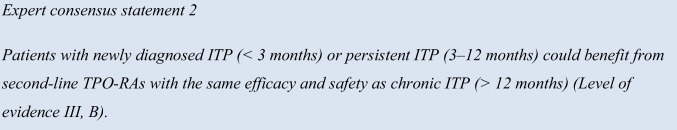


### Switching to an alternative TPO-RA

Data from the literature suggest that switching from one TPO-RA to another may positively affect response and tolerability. Five retrospective studies [42–46], one observational study [47], and two expert opinion articles [12, 31] were retrieved from the literature search to provide evidence for the possible beneficial effects of switching to an alternative TPO-RA. The experts reached 100% consensus (strongly agree *n* = 7, agree *n* = 1) for switching to an alternative TPO-RA if a patient with chronic ITP failed to respond/lost response, experienced adverse events, had platelet fluctuation, or due to patient inconvenience to their previous TPO-RA. Due to the lack of head-to-head randomized trials, the experts could not recommend a preferred TPO-RA for switching. However, 87.5% agreed (*n* = 7) that avatrombopag may be the preferred second TPO-RA agent to switch to if not used previously, based on the retrospective multicenter study recently reported by Al-Samkari et al. (2022) [46]. This study showed that 93% of patients switching from eltrombopag or romiplostim to avatrombopag achieved a platelet response (≥ 50 × 10^9^/L) and 86% achieved a complete response (CR) (≥ 100 × 10^9^/L) [46]. Furthermore, 57% of patients receiving concomitant ITP medications before switching discontinued them after switching to avatrombopag, including 63% of patients receiving chronic corticosteroids [46].
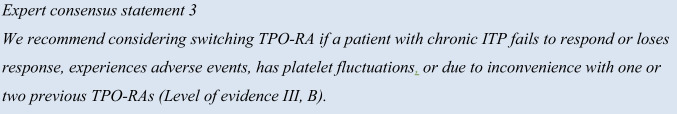


### Discontinuation of TPO-RAs

There is limited clinical guidance on the tapering/discontinuation of TPO-RAs in the literature [31]. Therefore, most recommendations are based only on expert opinion and real-world clinical experience. Six expert opinion reports were retrieved from the literature that provided recommendations for dose tapering TPO-RA regimens [12, 21, 31, 40, 41, 48]. The experts reached 75% consensus for dose tapering of TPO-RA regimens with possible discontinuation for individual ITP patients achieving sustained platelet counts above 50 × 10^9^/L (i.e., partial response [PR]) and 87.5% consensus for dose tapering above 100 × 10^9^/L (i.e., complete response, CR), and no bleeding for at least 12 months with TPO-RAs in the absence of other concomitant treatments.
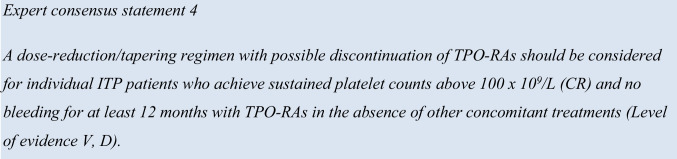


## Preferred TPO-RAs for specific patient populations

### Specific dietary requirements

Product information for the three approved TPO-RAs provides guidance on how each agent should be administered [17, 19, 29, 49]. Notably, eltrombopag should be taken at least 2 h before or 4 h after any products such as antacids, dairy products, or mineral supplements containing polyvalent cations to avoid significant reduction in eltrombopag absorption due to chelation [18]. Notably, romiplostim [17] and avatrombopag [19] have no such administration restrictions. In addition, romiplostim is the only parenteral TPO-RA that might be suitable for patients with gastrointestinal impairments (diarrhea, vomiting, GI surgery, etc.) [17]. One meta-analysis and one open-label RCT were retrieved from the literature search to support this consensus statement [29, 49]. The experts reached 100% consensus (strongly agree *n* = 2, agree *n* = 6) for recommending second-line romiplostim or avatrombopag as preferable to eltrombopag for ITP patients with specific dietary requirements (e.g., dairy-free).
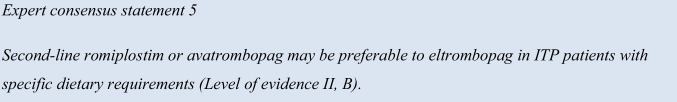


### Chronic ITP with concomitant liver dysfunction

In the product information for eltrombopag, the manufacturer reports a special caution regarding an increased risk of hepatic decompensation [18]. For romiplostim, the manufacturer reports cases of portal vein thrombosis in patients with chronic liver disease (CLD) who are receiving romiplostim and recommends that romiplostim be used with caution in these populations [17]. For avatrombopag, the manufacturer reports a very low risk of liver side effects based on results from two phase 3 studies, ADAPT-1 and ADAPT-2 [19]. Three RCTs [50–52], one exploratory trial [53], and one expert opinion report [54] were retrieved from the literature search to support the preference for TPO-RAs with low or very low risk of hepatic side effects in ITP patients with concomitant liver dysfunction. The experts reached 100% consensus (agree* n* = 8) for second-line TPO-RAs with very low risk of hepatic side effects (e.g., avatrombopag) as preferable in patients with chronic ITP and concomitant liver dysfunction.
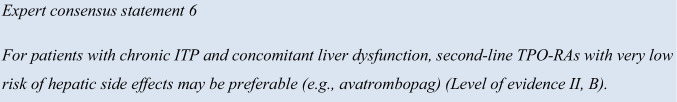


### ITP during pregnancy

Current international and ASH 2019 ITP guidelines do not promote the use of TPO-RAs during pregnancy [12, 13]; notably, pregnant patients were excluded from clinical trials evaluating TPO-RAs in adults. One retrospective study by Michel et al. (2020) retrieved from the literature search suggests that temporary off-label use of TPO-RAs for severe and/or refractory ITP during pregnancy benefits both mother and neonate, especially before delivery [58]. The experts all agreed (100% consensus) that TPO-RAs might be (rarely) considered for temporary off-label usage in individual ITP patients during late-stage pregnancy (e.g., for patients with severe, refractory ITP or contraindication to steroids and IVIG).
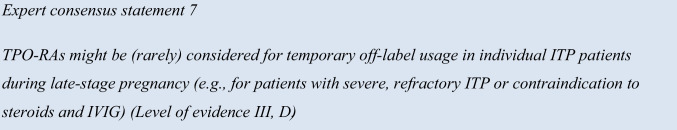


### Considerations for chronic ITP patients with mild/moderate COVID-19 

There are limited data on the impact of severe acute respiratory syndrome coronavirus 2 (SARS-CoV-2) infection (COVID-19) in patients previously diagnosed with primary ITP on TPO-RAs. Only one prospective study [55], one observational study [56], and one expert opinion article [57] related to the management of ITP during COVID-19 were retrieved from the literature search. Therefore, the experts could not make robust recommendations for chronic ITP patients with mild/moderate COVID-19. Approximately one-third of the experts (37.5%) did agree to the possibility of switching to another TPO-RA in the case of relapse for chronic ITP patients already on TPO-RA therapy with newly identified mild/moderate COVID-19 (three agreed, four neither agreed nor disagreed, one disagreed).

### Considerations for combined TPO-RA plus glucocorticoid therapy in first-line treatment of ITP

There is a lack of evidence in the literature demonstrating the utility of TPO-RA in the first-line setting [59–61]. One prospective study [59] and two proof-of-concept studies [60, 61] showed that TPO-RAs plus dexamethasone might be a possible frontline therapy for ITP. The experts discussed the combination of TPO-RA plus glucocorticoid therapy in the first-line setting. Less than two-thirds (62.5%) of the experts agreed that the potential combination of TPO-RA plus glucocorticoid therapy in the first-line setting might increase early remission rates and lower the likelihood of progression to chronic ITP.

## Discussion

These current consensus statements aim to provide guidance by reviewing the emerging literature and providing expert opinion specific to using TPO-RAs in adult ITP to help address real-world clinical practice issues. We found 40 relevant publications from the literature to support clinical decision-making in the adult ITP population, including five meta-analyses comprising a total of 31 unique RCTs. Consensus (> 75% agreement) was reached on seven statements for the second-line use of TPO-RAs in the management of adult ITP patients (Table 3).

**Table 3:** Summary of expert consensus statements on the use of TPO-RAs in adult ITP

1	Approved TPO-RAs (eltrombopag, romiplostim, or avatrombopag) are the preferred second-line treatment for chronic ITP patients who are refractory to a previous treatment (e.g., corticosteroids or immunoglobulins)
2	Consider TPO-RAs for newly diagnosed ITP (< 3 months) or persistent ITP (3–12 months)
3	Consider switching TPO-RA if a patient with chronic ITP fails to respond, loses response, or due to inconvenience, platelet fluctuations, or adverse events with one or two previous TPO-RAs
4	Consider a dose-reduction/tapering regimen with possible discontinuation of TPO for individual ITP patients with sustained platelet counts above 100 × 10^9^/L (CR) with TPO-RAs and no bleeding for at least 12 months in the absence of other concomitant treatments
5	Romiplostim or avatrombopag may be preferable to eltrombopag for specific ITP patients with dietary requirements
6	TPO-RAs with very low risk of hepatic side effects may be preferable (e.g., avatrombopag) for patients with chronic ITP and concomitant liver dysfunction
7	TPO-RAs might be (rarely) considered for temporary off-label usage in individual ITP patients during late-stage pregnancy (e.g., for patients with severe, refractory ITP or contraindication to steroids and IVIG)

The expert panel reached a 100% consensus on using TPO-RAs as the preferred second-line treatment for chronic ITP patients who are refractory to a previous treatment, such as corticosteroids or immunoglobulins [17–19]. The use of second-line TPO-RAs before 3 months after ITP diagnosis is not recommended in current international and ASH 2019 treatment guidelines [12, 13, 40]. Unsurprisingly, experts’ opinions on whether to avoid using TPO-RAs before 3 months of ITP were variable [12, 24, 39], suggestive of differing views regarding strict adherence to existing guidelines on managing ITP patients. However, for persistent ITP (≥ 3–12 months) in adults, both the ICR and ASH 2019 guidelines state that TPO-RAs can be used as second-line treatment in patients who are corticosteroid-dependent or unresponsive to corticosteroids [12, 13]. In addition, in adults with newly diagnosed ITP, the ASH guideline panel recommends against a prolonged course (> 6 weeks, including treatment and taper) of prednisone in favor of a short course (≤ 6 weeks) [13]. The ICR 2019 recommend stopping corticosteroids for newly diagnosed adult ITP by 6 weeks (maximum 8 weeks) [12]. Therefore, some ITP patients will need second-line treatment (with TPO-RAs or other) before 3 months of duration of ITP. In addition, it is important to mention that different TPO-RAs (eltrombopag, romiplostim, and avatrombopag) have different labels/official approvals regarding ITP duration in adults, which differ for the EMA and FDA [62]. For example, romiplostim and more recently eltrombopag have an EMA indication for the treatment of primary ITP in adult patients who are refractory to other treatments (e.g., corticosteroids, immunoglobulins) with no time restrictions [17, 18]. In contrast, avatrombopag is currently indicated for primary chronic ITP in adult patients who are refractory to other treatments (e.g., corticosteroids, immunoglobulins) [19, 62]. In addition, avatrombopag is also indicated for the treatment of severe thrombocytopenia in adults with CLD who are scheduled to undergo an invasive procedure [19, 62]. Furthermore, the Central European experts highlight the need for an individualized treatment approach. As increasing evidence becomes available on the effective use of early TPO-RAs in newly diagnosed ITP patients (i.e., ITP less than 3 months of duration), recommendations in future guidelines for treating ITP earlier in the second-line setting are likely to be revised.

ITP is a common cause of low platelet count (below 50 × 10^9^/L) during the first and second trimesters of pregnancy, accounting for about 3% of all thrombocytopenia cases [63]. Various studies show that up to 35% of affected mothers may require treatment even prior to the management of labor and delivery [63]. Corticosteroids and IVIGs are commonly used to treat acute ITP during pregnancy, but TPO-RAs are an attractive alternative [64]. However, none of the trials evaluating the use of TPO-RAs in adults with ITP included pregnant patients or lactating mothers [64]. Moreover, current literature evidence demonstrating the use of TPO-RAs in pregnancy is primarily limited to off-label use in individual patients [64]. One recent review suggests that TPO-RAs can help raise the platelet count within 2–3 weeks in pregnant patients with ITP [64]. The experts in this study agreed that in rare situations, temporary off-label use of TPO-RAs for severe and/or refractory ITP in pregnant women might be considered during late-stage pregnancy, i.e., even before delivery. Notably, TPO-RA use during the first trimester of pregnancy, i.e., when organogenesis is at its peak, must be avoided until studies demonstrating fetal and maternal safety become available [64].

In addition to the seven consensus statements, the experts also considered and discussed TPO-RA treatment in two further patient populations but failed to reach a consensus: chronic ITP patients with mild/moderate COVID-19 and treatment of ITP patients in the first-line setting. Hematologic complications of COVID-19 have been reported, including ITP secondary to COVID-19; however, the mechanisms involved remain unknown [65, 66]. Due to the increased potential for thrombotic complications and hepatotoxicity, interim COVID-19 guidance suggests using TPO-RAs only as a second-line agent in COVID-19 patients with no evidence of disseminated intravascular coagulation (DIC) [67]. Current ITP guidelines also recommend that patients with chronic ITP remain on their usual treatment if they test positive for COVID-19 [68]. In a comprehensive review of reported cases in the literature, Berger and Rodgers (2021) concluded that treatment regimens including TPO-RAs are most effective for obtaining a complete response, and steroids may be more effective than IVIG in patients with ITP secondary to COVID-19 [66]. An earlier systematic review by Bhattacharjee and Banerjee reported that no adverse effects were observed with a short duration of TPO-RA as second-line therapy in a few COVID-19 cases (*n* = 9) [65]. Furthermore, there are very few reports of ITP exacerbation following COVID-19 vaccine administration [69]. Given the limited cases, there are currently no guidelines for managing ITP caused by the COVID-19 vaccine or vaccination of individuals with predisposing conditions [69]. Unsurprisingly, the experts in this study did not concur on the possibility of switching to another TPO-RA for chronic ITP patients in case of relapse already on TPO-RA therapy with newly identified mild/moderate COVID-19. Further studies on using TPO-RAs in adult ITP patients with mild/moderate COVID-19 are required.

Platelet responses following TPO-RA treatment usually decrease gradually once medications are stopped, and questions on whether the combination of TPO-RAs with other drugs (e.g., corticosteroids) can exert additive effects and provide better clinical benefits than TPO-RAs alone have been raised [70]. However, there is a lack of data demonstrating the utility of TPO-RA combination therapy in the first-line setting, and no expert recommendations can be made at present. Corticosteroids are a mainstay first-line treatment of ITP but are frequently overused and associated with limiting toxicities [71]. Reductions in corticosteroid use may improve health-related quality of life in patients with ITP [72]. The experts concurred that TPO-RAs plus glucocorticoids could provide a promising first-line therapy with a dual action (i.e., immunosuppression and stimulation of platelet production) with the potential to decrease corticosteroid exposure for a significant number of ITP adults. Yu et al. (2020) showed that combination of TPO‐RA therapy plus corticosteroid therapy results in a higher incidence of initial response (89.0% vs 66.7%, *P* < 0.001) and CR (75.0% vs 42.7%, *P* < 0.001) compared with corticosteroids alone [59].

Due to a lack of head-to-head studies and evidence for specific ITP populations and scenarios, the consensus statements provided in this paper are based on expert opinion as well as on literature data. Therefore, we have drawn on evidence from clinical trials in the literature, where possible. However, we acknowledge the possibility of intra-publication bias and homogeneity of expert opinion. We believe that the consensus statements are applicable throughout the international ITP community; however, the information may be less transferable in nations not represented herein.

## Conclusions

The findings from this systematic literature review have informed the development of consensus statements by a group of senior ITP hematologists from eight countries across Central Europe. The consensus statements aim to raise awareness, provide guidance, and facilitate informed decision-making on key issues that healthcare providers must consider when using TPO-RAs to treat their adult ITP patients in real-life practice. The expert panel achieved a high level of agreement on seven statements, including earlier usage of TPO-RA as a second-line treatment in ITP and switching and tapering TPO-RAs. In general, an individualized approach to managing adults with ITP is strongly recommended, taking into account patient preferences, comorbidities, and lifestyle. There are considerations with a lack of expert consensus in this work. Updated treatment guidelines will therefore be required if adequate new studies of TPO-RAs in the first-line setting or in specific patient groups such as COVID-19 ITP patients become available.

## Supplementary Information

Below is the link to the electronic supplementary material.

Supplementary file1 (DOCX 88 KB)
