# Supplementary material for: Use of thrombopoietin receptor agonists in adults with immune thrombocytopenia: a systematic review and Central European expert consensus
Source: Ann Hematol. 2023 Feb 24;102(4):715–27. doi: 10.1007/s00277-023-05114-8 (PMC9951167; doi:10.1007/s00277-023-05114-8)
Supplement: Supplementary file 1 — Supplementary file1 (DOCX 88 KB) [file 277_2023_5114_MOESM1_ESM.docx]

# Table S1. Characteristics and quality of evidence of included studies

| **Author (year)** | **Study type** | **TPO-RA(s)/treatment; line of therapy** | **Study population (Sample size)** | **Primary outcome measure** | **Key Conclusions(s)** | **Quality of evidence (OCEBM)** |
| --- | --- | --- | --- | --- | --- | --- |
| Afdhal et al. (2012)[1] | Phase 3, international, double-blind, placebo-controlled RCT | Eltrombopag vs placebo | Adults with thrombocytopenia and CLD undergoing an elective invasive procedure (n=292) | Avoidance of a platelet transfusion before, during, and up to 7 days after the procedure | Eltrombopag reduced the need for platelet transfusions in patients with CLD who were undergoing elective invasive procedures, but it was associated with an increased incidence of portal-vein thrombosis, as compared with placebo | I |
| Birocchi et al. (2021)[2] | SR and MA of 16 RCTs and 19 cohort studies published until 2018 | Eltrombopag, romiplostim, avatrombopag; 2L | Primary ITP patients (RCTs: n=909 primary ITP patients assigned to TPO-RA and n=427 to the control arm) | Risk ratio of treatment failure and bleeding of WHO grade ≥2; rate of remission after discontinuation of treatment | TPO-RAs are effective and safe in patients with ITP, even in the long term | I |
| Cohen et al. (2021)[3] | MA of 6 RCTs | Targeted therapies: eltrombopag romiplostim, avatrombopag, fostamatinib; 2L | ITP patients (n=752) | Platelet recovery to ≥ 50,000/µL, major and minor bleeding events, and survival | Compared to placebo, targeted therapies for ITP increase platelet counts, decrease bleeding events and show a trend towards lower mortality without increased toxicity | I |
| Deng et al. (2021)[4] | MA of 20 RCTs | Avatrombopag, lusutrombopag, eltrombopag, romiplostim, rhTPO; 2L | ITP patients (n=2,207) | The number of patients achieving platelet response which was defined as the achievement of a platelet count of more than 30 or 50 cells × 10^9^/L in the absence of rescue therapy | Avatrombopag may yield the highest efficacy because it has the most favorable balance of benefits and acceptability. | I |
| Terrault et al. (2018)[5] | Two phase 3, international, multicenter, double-blind, placebo-controlled RCTs | Avatrombopag vs placebo | Adults with CLD and thrombocytopenia with a mean baseline platelet count of < 50 x 10^9^/L (n=435) | Proportion of patients not requiring platelet transfusions or rescue procedures for bleeding up to 7 days after a scheduled procedure | Avatrombopag was superior to placebo in reducing the need for platelet transfusions or rescue procedures for bleeding in patients with thrombocytopenia and CLD undergoing a scheduled procedure | I |
| Wojciechowski et al. (2021)[6] | MA of 7 RCTs | Eltrombopag, romiplostim, avatrombopag, fostamatinib; 2L | Chronic ITP (n=470) | Durable platelet response; need for rescue treatment; WHO grade 2–4 bleeding events | Avatrombopag significantly increased the chance of achieving durable platelet response and reducing the use of concomitant ITP medication vs. placebo, and significantly reduced the incidence of any bleeding events compared with placebo, eltrombopag, and romiplostim | I |
| Yang et al. (2019)[7] | SR and MA of 13 RCTs | Eltrombopag, romiplostim, avatrombopag, fostamatinib, rituximab; 2L | ITP (n=1,202) | Overall response, defined as a platelet count ≥50 × 10^9^/L at the end of treatment without rescue therapy), while the secondary endpoints included early response (i.e., a platelet count ≥50 × 10^9^/L at week 2 after initiation of treatment) and therapy-related severe AEs | Romiplostim appears to be the best option for patients who fail to respond to prior treatment or relapse thereafter, while avatrombopag and eltrombopag are reasonable alternatives; Rituximab monotherapy is not recommended, as it produces the lowest overall response and early response rates. | I |
| Newland et al. (2016)[8] | Phase 2, single arm, multicenter prospective study | Romiplostim; 2L | Adults with early-stage primary ITP (n=75); Median (Q1, Q3) ITP duration before enrolment was 2.2 (0.9, 4.3) months | Cumulative number of months in which patients achieved a platelet response (≥50 x 10^9^/L) during the 12-month treatment period | In patients with early-stage ITP, romiplostim was well tolerated and induced rapid responses, with remission occurring in approximately one-third of patients | II |
| Terrault et al. (2014)[9] | Phase 2, multicenter, double-blind, placebo-controlled, RCT | Avatrombopag vs placebo | Adults with CLD and thrombocytopenia undergoing an elective procedure (n=93) | Increase in platelet count ≥20 x 10^9^/L above baseline and at least one platelet count >50 x 10^9^/L from days 4–8 | Avatrombopag was generally well-tolerated and increased platelet counts in patients with cirrhosis undergoing elective invasive procedures | II |
| Wire et al. (2012)[10] | OL, crossover RCT | Eltrombopag | Healthy adults (n=40) | Tolerability was assessed by laboratory tests, physical examinations, and AEs | In a healthy adult volunteer population, bioavailability of eltrombopag PfOS was reduced when administered with or 2 hours before or after a high-calcium meal; this effect was attenuated with PfOS dosing 2 hours before the meal. Eltrombopag was generally well tolerated | II |
| Yu et al. (2020)[11] | RCT (Prospective) | HD-DXM plus rhTPO, vs HD-DXM; 1L | Newly diagnosed Chinese adults with primary ITP (n=196) | Initial response and sustained response | The combination of HD-DXM with rhTPO significantly improved the initial response and yielded favorable sustained response in newly diagnosed ITP patients, thus could be further validated as a frontline treatment for ITP | II |
| Al-Samkari et al. (2022)[12] | Retrospective study | Romiplostim and/or eltrombopag and/or avatrombopag; 2L | ITP patients on TPO-RA who underwent switch (n=44) | Treatment response (achievement of a given platelet count on at least one occasion and without requirement for rescue therapy), use of concomitant ITP medications, AEs with TPO-RAs before and after switching | In a heavily pretreated chronic ITP population, avatrombopag was effective following therapy with romiplostim or eltrombopag, with high response rates even in patients with inadequate response to a prior TPO-RA | III |
| Cantoni et al. (2018)[13] | Retrospective study | Eltrombopag and/or romiplostim; 2L | ITP patients on TPO-RA who underwent switch (n=546) | TPO-RA sequence; adverse events; outcome and follow-up after switching | Efficacy of TPO-RA switch; once achieved, response to the 2nd TPO-RA seems durable | III |
| Gómez-Almaguer et al. (2014)[14] | Single-arm, open-label proof-of-concept study | Eltrombopag, dexamethasone combination; 1L | Newly diagnosed ITP (n=12) | End-of-treatment (day 33) response rate | Eltrombopag/dexamethasone is a feasible frontline therapy for ITP | III |
| González-López et al. (2017)[15] | Retrospective study | Eltrombopag; 2L | Adult primary ITP (n=220; newly diagnosed n=30, persistent n=30, chronic n=160) | Effectiveness outcomes and safety | Eltrombopag use for early-stage ITP is considered as effective and safe as it is in chronic ITP | III |
| González-Porras et al. (2014)[16] | Retrospective study | Eltrombopag after romiplostim; 2L | ITP patients were sequentially treated first with romiplostim and then with eltrombopag (n=51) | Efficacy and tolerance of using eltrombopag after romiplostim | Use of eltrombopag after romiplostim for treating ITP to be effective and safe | III |
| Khellaf et al. (2013)[17] | Retrospective pilot study | Eltrombopag and/or romiplostim; 2L | ITP patients on TPO-RA who underwent switch (n=46) | Sustained response and AEs | Switching from one TPO-RA to the other could be beneficial in clinical practice for patients with severe chronic ITP who failed to respond or experienced adverse events to the first | III |
| Kurokawa et al. (2016)[18] | Exploratory clinical study | Eltrombopag | Patients with CLD and hepatitis C virus infection who presented with thrombocytopenia but without cancer (n=5) | Clinical and biological data | Results indicate the safety of long-term eltrombopag administration for patients with CLD and hepatitis C virus infection | III |
| Kuter et al. (2015)[19] | Observational study | Eltrombopag and/or romiplostim; 2L | ITP patients who underwent a switch to TPO/RA (n=280) | The rationale for switching to TPO-RA, dosing patterns, treatment outcomes | Switching to the other TPO-RA may be beneficial if there is an inadequate response to treatment with the initial TPO-RA. | III |
| Kuter et al. (2019)[20] | Exploratory retrospective analysis | Romiplostim; 2L | Patients with ITP for ≤1 year (n=311) or >1 year (n=726) | Effect of romiplostim in each ITP duration subgroup for platelet response, bleeding and AEs, including thrombosis | Romiplostim increased platelet counts in patients with either ITP ≤1 year or ITP >1 year, with more treatment-free remission in those with ITP ≤1 year | III |
| Lakhwani et al. (2017)[21] | Retrospective analysis | Eltrombopag and/or romiplostim; 2L | ITP patients on TPO-RA who underwent switch (n=26) | Clinical and biological parameters | TPO-RA switching is a feasible strategy in different scenarios with a high probability of success | III |
| Meyer et al. (2021)[22] | Retrospective study | Romiplostim and/or eltrombopag; 2L | Patients receiving ≥1 TPO-RA prescriptions (n=3,553) | ITP medication use was assessed for 18 months prior to, during and for ≥6 months after TPO-RA treatment. | Earlier use of TPO-RA therapy after a short course of corticosteroids could avoid side effects associated with long-term use | III |
| Michel et al. (2020)[23] | Multicenter, retrospective, observational study | Eltrombopag or romiplostim | Pregnant women with ITP (n=15; pregnancies n=17, neonates n=18) | Clinical and biological data; treatment response (complete response) | Transient use of eltrombopag or romiplostim for pregnant women with ITP who are refractory to at least corticosteroids and IV immunoglobulin and need treatment because of bleeding manifestations, because of profound thrombocytopenia, and/or in preparation for delivery seems safe for the mother and the fetus/neonate | III |
| Mingot-Castellano et al. (2018)[24] | Retrospective study | Romiplostim and/or eltrombopag; | ITP patients n=100 treated with TPO-RAs (n=122) (chronic n=97, persistent n=12 and newly diagnosed n=13) | Experience with TPO-RAs in ITP (chronic, persistent and newly diagnosed ITP) in routine clinical practice | TPO-RAs may be useful in all types of ITP, not only chronic but also persistent and newly diagnosed | III |
| Moulis et al. (2022)[25] | Real-world prospective study | Eltrombopag; 2L | Adults with ITP (n=156); ITP duration <6 months (n=95) | Effectiveness outcomes and safety | In the French real-world practice, Eltrombopag was used early (off-label) in the ITP course. The effectiveness and safety profiles identified in clinical trials and previous retrospective real-world series were confirmed, even in the subgroup of disease duration <6 months | III |
| Pantic et al. (2022)[26] | Prospective/ and retrospective study | Eltrombopag or romiplostim | Chronic ITP patients with COVID-19 (n=7) | Data regarding the clinical course of COVID-19 were collected | Careful platelet count monitoring and therapy management are needed in chronic ITP patients on TPO-RAs with COVID-19 | III |
| Snell-Taylor et al. (2021)[27] | Post-hoc analysis of an international observational study | Romiplostin; 2L | ITP patients (n=100); newly diagnosed (n=22), persistent (n=17), chronic (n=61) | To assess the effectiveness and safety of romiplostim within 24 weeks after initiation by duration of ITP: ‘newly diagnosed,’ ‘persistent,’ and ‘chronic’ | Romiplostim was effective and well tolerated, irrespective of ITP duration at the time of romiplostim initiation. Prescribing romiplostim in early ITP was considered off-label at the time of the conduct of the study | III |
| Tripathi et al. (2014)[28] | Single center retrospective study | Eltrombopag; 1L | Newly diagnosed steroid non-responsive ITP patients (n=25) | Platelet response | Results suggest a possible role of eltrombopag in newly diagnosed steroid non-responsive ITP patients | III |
| Visser et al. (2022)[29] | Multicenter observational study | Eltrombopag, romiplostim, glucocorticoids, rituximab | Adult ITP patients newly diagnosed, persistent, chronic, remission) who received >1 COVID-19 vaccination (n=218) and healthy controls (n=200) | To study platelet dynamics in patients with ITP after COVID-19 vaccination | In a subgroup of patients with ITP, close monitoring of platelet counts with COVID-19 vaccination is recommended | III |
| Zhang et al. (2020)[30] | Single-arm, open-label pilot study | Eltrombopag/dexamethasone combination; 1L | ITP patients (n=50) | Durable response off-therapy defined as maintaining platelet counts >50 × 10^9^/L for more than six months without further ITP therapy | Eltrombopag plus pulsed dexamethasone as a 1L therapy could result in a durable response off-therapy in a significant number of ITP subjects | III |
| Zhang and Miao (2021)[31] | Retrospective analysis | Eltrombopag; 2L | ITP patients (n=85) | Eltrombopag-induced liver dysfunction | The risk of early liver dysfunction, while mild to moderate in most cases, is high in patients with ITP on eltrombopag treatment, especially in those with type 2 diabetes and hepatobiliary diseases | III |
| Carpenedo et al. (2021)[32] | Expert opinion – Italian Delphi consensus | Eltrombopag, romiplostim | na | na | na | V |
| Cooper et al. (2021)[33] | Expert opinion –Delphi consensus | Eltrombopag, romiplostim | na | na | na | V |
| Cuker et al. (2020)[34] | Expert opinion –Delphi consensus | Eltrombopag, romiplostim, avatrombopag | na | na | na | V |
| Desouza et al. (2022)[35] | Expert opinion – ASH guidelines | Eltrombopag, romiplostim | na | na | na | V |
| Flisiak et al. (2021)[36] | Expert opinion - CEHE | Avatrombopag | na | na | na | V |
| Matzdorff et al. (2018)[37] | Expert view – DGHO recommendations | Eltrombopag, romiplostim | na | na | na | V |
| Neunert et al. (2019)[38] | Expert opinion – ASH guidelines | Eltrombopag, romiplostim | na | na | na | V |
| Provan et al. (2019)[39] | Expert opinion – ICR consensus | Eltrombopag, romiplostim, avatrombopag | na | na | na | V |
| Sahu et al. (2020)[40] | Expert opinion | Eltrombopag, romiplostim, avatrombopag | na | na | na | V |

Abbreviations: 1L, first line; 2l, second line; AEs, adverse events; ASH, American Society of Hematology; CEHE, Central European Hepatologic Collaboration; CLD, chronic liver disease; DGHO, German Society for Hematology and Medical Oncology; HD-DXM, high-dose dexamethasone; ICR, International Consensus Report; ITP, immune thrombocytopenia; IV, intravenous; na, not applicable; PfOS, powder for oral suspension; MA, meta-analysis; Q, quartile; RCT, randomized controlled trial; rhTPO, recombinant human thrombopoietin; SR, systematic review; TPO-RA, thrombopoietin receptor agonist; WHO, World Health Organization.

**References**

1. Afdhal NH, Giannini EG, Tayyab G, Mohsin A, Lee JW, Andriulli A, Jeffers L, McHutchison J, Chen PJ, Han KH, Campbell F, Hyde D, Brainsky A, Theodore D (2012) Eltrombopag before procedures in patients with cirrhosis and thrombocytopenia. N Engl J Med 367 (8):716-724. <https://doi.org/10.1056/NEJMoa1110709>

2. Birocchi S, Podda GM, Manzoni M, Casazza G, Cattaneo M (2021) Thrombopoietin receptor agonists for the treatment of primary immune thrombocytopenia: a meta-analysis and systematic review. Platelets 32 (2):216-226. <https://doi.org/10.1080/09537104.2020.1745168>

3. Cohen I, Goldvaser H, Kirgner I, Leader A, Raanani P, Isakov O, Shepshelovich D (2021) Targeted therapies for immune thrombocytopenic purpura: a meta-analysis of randomized controlled trials. Ann Hematol 100 (12):2879-2887. <https://doi.org/10.1007/s00277-021-04669-8>

4. Deng J, Hu H, Huang F, Huang C, Huang Q, Wang L, Wu A, Yang J, Qin D, Zou W, Wu J (2021) Comparative Efficacy and Safety of Thrombopoietin Receptor Agonists in Adults With Thrombocytopenia: A Systematic Review and Network Meta-analysis of Randomized Controlled Trial. Front Pharmacol 12:704093. <https://doi.org/10.3389/fphar.2021.704093>

5. Terrault N, Chen YC, Izumi N, Kayali Z, Mitrut P, Tak WY, Allen LF, Hassanein T (2018) Avatrombopag Before Procedures Reduces Need for Platelet Transfusion in Patients With Chronic Liver Disease and Thrombocytopenia. Gastroenterology 155 (3):705-718. <https://doi.org/10.1053/j.gastro.2018.05.025>

6. Wojciechowski P, Wilson K, Nazir J, Pustułka I, Tytuła A, Smela B, Pochopień M, Vredenburg M, McCrae KR, Jurczak W (2021) Efficacy and Safety of Avatrombopag in Patients with Chronic Immune Thrombocytopenia: A Systematic Literature Review and Network Meta-Analysis. Advances in Therapy 38:3113 - 3128. <https://doi.org/10.1007/s12325-021-01752-4>

7. Yang R, Lin L, Yao H, Ji O, Shen Q (2019) Therapeutic options for adult patients with previously treated immune thrombocytopenia - a systematic review and network meta-analysis. Hematology 24 (1):290-299. <https://doi.org/10.1080/16078454.2019.1568659>

8. Newland A, Godeau B, Priego V, Viallard JF, López Fernández MF, Orejudos A, Eisen M (2016) Remission and platelet responses with romiplostim in primary immune thrombocytopenia: final results from a phase 2 study. Br J Haematol 172 (2):262-273. <https://doi.org/10.1111/bjh.13827>

9. Terrault NA, Hassanein T, Howell CD, Joshi S, Lake J, Sher L, Vargas H, McIntosh J, Tang S, Jenkins TM (2014) Phase II study of avatrombopag in thrombocytopenic patients with cirrhosis undergoing an elective procedure. J Hepatol 61 (6):1253-1259. <https://doi.org/10.1016/j.jhep.2014.07.007>

10. Wire MB, Bruce J, Gauvin J, Pendry CJ, McGuire S, Qian Y, Brainsky A (2012) A Randomized, Open-Label, 5-Period, Balanced Crossover Study to Evaluate the Relative Bioavailability of Eltrombopag Powder for Oral Suspension (PfOS) and Tablet Formulations and the Effect of a High-Calcium Meal on Eltrombopag Pharmacokinetics When Administered With or 2 Hours Before or After PfOS. Clinical Therapeutics 34 (3):699-709. <https://doi.org/10.1016/j.clinthera.2012.01.011>

11. Yu Y, Wang M, Hou Y, Qin P, Zeng Q, Yu W, Guo X, Wang J, Wang X, Liu G, Chu X, Yang L, Feng Y, Zhou F, Sun Z, Zhang M, Wang X, Wang Z, Ran X, Zhao H, Wang L, Zhang H, Bi K, Li D, Yuan C, Xu R, Wang Y, Zhou Y, Peng J, Liu XG, Hou M (2020) High-dose dexamethasone plus recombinant human thrombopoietin vs high-dose dexamethasone alone as frontline treatment for newly diagnosed adult primary immune thrombocytopenia: A prospective, multicenter, randomized trial. Am J Hematol 95 (12):1542-1552. <https://doi.org/10.1002/ajh.25989>

12. Al-Samkari H, Jiang D, Gernsheimer T, Liebman H, Lee S, Wojdyla M, Vredenburg M, Cuker A (2022) Adults with immune thrombocytopenia who switched to avatrombopag following prior treatment with eltrombopag or romiplostim: A multicentre US study. British Journal of Haematology 197 (3):359-366. <https://doi.org/10.1111/bjh.18081>

13. Cantoni S, Carpenedo M, Mazzucconi MG, De Stefano V, Carrai V, Ruggeri M, Specchia G, Vianelli N, Pane F, Consoli U, Artoni A, Zaja F, D'Adda M, Visentin A, Ferrara F, Barcellini W, Caramazza D, Baldacci E, Rossi E, Ricco A, Ciminello A, Rodeghiero F, Nichelatti M, Cairoli R (2018) Alternate use of thrombopoietin receptor agonists in adult primary immune thrombocytopenia patients: A retrospective collaborative survey from Italian hematology centers. Am J Hematol 93 (1):58-64. <https://doi.org/10.1002/ajh.24935>

14. Gómez-Almaguer D, Herrera-Rojas MA, Jaime-Pérez JC, Gómez-De León A, Cantú-Rodríguez OG, Gutiérrez-Aguirre CH, Tarín-Arzaga L, Hernández-Reyes J, Ruiz-Arguelles GJ (2014) Eltrombopag and high-dose dexamethasone as frontline treatment of newly diagnosed immune thrombocytopenia in adults. Blood 123 (25):3906-3908. <https://doi.org/10.1182/blood-2014-01-549360>

15. González-López TJ, Fernández-Fuertes F, Hernández-Rivas JA, Sánchez-González B, Martínez-Robles V, Alvarez-Román MT, Pérez-Rus G, Pascual C, Bernat S, Arrieta-Cerdán E, Aguilar C, Bárez A, Peñarrubia MJ, Olivera P, Fernández-Rodríguez A, de Cabo E, García-Frade LJ, González-Porras JR (2017) Efficacy and safety of eltrombopag in persistent and newly diagnosed ITP in clinical practice. International Journal of Hematology 106 (4):508-516. <https://doi.org/10.1007/s12185-017-2275-4>

16. González-Porras JR, Mingot-Castellano ME, Andrade MM, Alonso R, Caparrós I, Arratibel MC, Fernández-Fuertes F, Cortti MJ, Pascual C, Sánchez-González B, Bernat S, Fuertes-Palacio MA, Vázquez-Paganini JA, Olivera PE, Alvarez-Román MT, Jarque I, Cortés M, Martínez-Robles V, Díaz-Gálvez FJ, Calbacho M, Fernández-Miñano C, Garcia-Frade J, González-López TJ (2015) Use of eltrombopag after romiplostim in primary immune thrombocytopenia. British Journal of Haematology 169 (1):111-116. <https://doi.org/10.1111/bjh.13266>

17. Khellaf M, Viallard JF, Hamidou M, Cheze S, Roudot-Thoraval F, Lefrere F, Fain O, Audia S, Abgrall JF, Michot JM, Dauriac C, Lefort S, Gyan E, Niault M, Durand JM, Languille L, Boutboul D, Bierling P, Michel M, Godeau B (2013) A retrospective pilot evaluation of switching thrombopoietic receptor-agonists in immune thrombocytopenia. Haematologica 98 (6):881-887. <https://doi.org/10.3324/haematol.2012.074633>

18. Kurokawa T, Murata S, Ohkohchi N (2016) Stable Liver Function during Long-Term Administration of Eltrombopag, a Thrombopoietin Receptor Agonist, in Patients with Chronic Liver Disease. Tohoku J Exp Med 240 (4):277-279. <https://doi.org/10.1620/tjem.240.277>

19. Kuter DJ, Macahilig C, Grotzinger KM, Poston SA, Wang PF, Dawson KL, Ward M (2015) Treatment patterns and clinical outcomes in patients with chronic immune thrombocytopenia (ITP) switched to eltrombopag or romiplostim. Int J Hematol 101 (3):255-263. <https://doi.org/10.1007/s12185-014-1731-7>

20. Kuter DJ, Newland A, Chong BH, Rodeghiero F, Romero MT, Pabinger I, Chen Y, Wang K, Mehta B, Eisen M (2019) Romiplostim in adult patients with newly diagnosed or persistent immune thrombocytopenia (ITP) for up to 1 year and in those with chronic ITP for more than 1 year: a subgroup analysis of integrated data from completed romiplostim studies. British journal of haematology 185 (3):503-513. <https://doi.org/10.1111/bjh.15803>

21. Lakhwani S, Perera M, Fernández-Fuertes F, Ríos de Paz MA, Torres M, Raya JM, Hernández MT (2017) Thrombopoietin receptor agonist switch in adult primary immune thrombocytopenia patients: A retrospective collaborative survey involving 4 Spanish centres. Eur J Haematol 99 (4):372-377. <https://doi.org/10.1111/ejh.12932>

22. Meyer O, Richter H, Lebioda A, Schill M (2021) Treatment patterns in adults with immune thrombocytopenia before, during and after use of thrombopoietin receptor agonists: a longitudinal prescription database study from Germany. Hematology 26 (1):697-708. <https://doi.org/10.1080/16078454.2021.1974203>

23. Michel M, Ruggeri M, Gonzalez-Lopez TJ, Alkindi S, Cheze S, Ghanima W, Tvedt THA, Ebbo M, Terriou L, Bussel JB, Godeau B (2020) Use of thrombopoietin receptor agonists for immune thrombocytopenia in pregnancy: results from a multicenter study. Blood 136 (26):3056-3061. <https://doi.org/10.1182/blood.2020007594>

24. Mingot-Castellano ME, Caparrós IS, Fernández F, Perera-Alvarez MDM, Jimenez-Bárcenas R, Casaus García A, González-Silva M, Yera-Cobo M, Nieto-Hernandez MM, Rodríguez-Fernandez MJ, Díaz-Canales D (2018) Treatment characteristics, efficacy and safety of thrombopoietin analogues in routine management of primary immune thrombocytopenia. Blood Coagul Fibrinolysis 29 (4):374-380. <https://doi.org/10.1097/mbc.0000000000000726>

25. Moulis G, Germain J, Rueter M, Lafaurie M, Aroichane M, Comont T, Mahévas M, Viallard JF, Chèze S, Ebbo M, Audia S, Leclerc-Teffahi S, Sommet A, Beyne-Rauzy O, Michel M, Godeau B, Lapeyre-Mestre M (2022) Eltrombopag in adult patients with immune thrombocytopenia in the real-world in France, including off-label use before 6 months of disease duration: The multicenter, prospective ELEXTRA study. Am J Hematol 97 (2):E40-e44. <https://doi.org/10.1002/ajh.26404>

26. Pantic N, Suvajdzic-Vukovic N, Virijevic M, Pravdic Z, Sabljic N, Adzic-Vukicevic T, Mitrovic M (2022) Coronavirus disease 2019 in patients with chronic immune thrombocytopenia on thrombopoietin receptor agonists: new perspectives and old challenges. Blood Coagulation & Fibrinolysis 33 (1):51-55. <https://doi.org/10.1097/MBC.0000000000001109>

27. Snell Taylor SJ, Nielson CM, Breskin A, Saul B, Yu Y, Alam N, Eisen M, Hippenmeyer J, Janssens A, Kozak T, Papadaki HA, Selleslag D, Viallard JF, Feistritzer C, Kaiafa G, Kelsh M, Kilpatrick K, Brookhart MA, McGrath LJ (2021) Effectiveness and Safety of Romiplostim Among Patients with Newly Diagnosed, Persistent and Chronic Immune Thrombocytopenia in European Clinical Practice. Adv Ther 38 (5):2673-2688. <https://doi.org/10.1007/s12325-021-01727-5>

28. Tripathi AK, Shukla A, Mishra S, Yadav YS, Yadav DK (2014) Eltrombopag therapy in newly diagnosed steroid non-responsive ITP patients. Int J Hematol 99 (4):413-417. <https://doi.org/10.1007/s12185-014-1533-y>

29. Visser C, Swinkels M, van Werkhoven ED, Croles FN, Noordzij-Nooteboom HS, Eefting M, Last-Koopmans SM, Idink C, Westerweel PE, Santbergen B, Jobse PA, Baboe F, Consortium R-I, Te Boekhorst PAW, Leebeek FWG, Levin M-D, Kruip MJHA, Jansen AJG (2022) COVID-19 vaccination in patients with immune thrombocytopenia. Blood advances 6 (6):1637-1644. <https://doi.org/10.1182/bloodadvances.2021006379>

30. Zhang L, Zhang M, Du X, Cheng Y, Cheng G (2020) Safety and efficacy of eltrombopag plus pulsed dexamethasone as first-line therapy for immune thrombocytopenia. Br J Haematol 189 (2):369-378. <https://doi.org/10.1111/bjh.16327>

31. Zhang P, Miao W (2021) Eltrombopag-induced liver dysfunction during the treatment of immune thrombocytopenia and its risk factors. Ann Palliat Med 10 (6):6419-6424. <https://doi.org/10.21037/apm-21-1067>

32. Carpenedo M, Baldacci E, Baratè C, Borchiellini A, Buccisano F, Calvaruso G, Chiurazzi F, Fattizzo B, Giuffrida G, Rossi E, Palandri F, Scalzulli PR, Siragusa SM, Vitucci A, Zaja F (2021) Second-line administration of thrombopoietin receptor agonists in immune thrombocytopenia: Italian Delphi-based consensus recommendations. Therapeutic Advances in Hematology 12:1-9. <https://doi.org/10.1177/20406207211048361>

33. Cooper N, Hill QA, Grainger J, Westwood JP, Bradbury C, Provan D, Thachil J, Ramscar N, Roy A (2021) Tapering and Discontinuation of Thrombopoietin Receptor Agonist Therapy in Patients with Immune Thrombocytopenia: Results from a Modified Delphi Panel. Acta Haematol 144 (4):418-426. <https://doi.org/10.1159/000510676>

34. Cuker A, Despotovic JM, Grace RF, Kruse C, Lambert MP, Liebman HA, Lyons RM, McCrae KR, Pullarkat V, Wasser JS, Beenhouwer D, Gibbs SN, Yermilov I, Broder MS (2020) Tapering thrombopoietin receptor agonists in primary immune thrombocytopenia: Expert consensus based on the RAND/UCLA modified Delphi panel method. Research and practice in thrombosis and haemostasis 5 (1):69-80. <https://doi.org/10.1002/rth2.12457>

35. DeSouza S, Angelini D (2021) Updated guidelines for immune thrombocytopenic purpura: Expanded management options. Cleveland Clinic Journal of Medicine 88 (12):664-668. <https://doi.org/10.3949/ccjm.88a.20201>

36. Flisiak R, Antonov K, Drastich P, Jarcuska P, Maevskaya M, Makara M, Puljiz Ž, Štabuc B, Trifan A (2021) Practice Guidelines of the Central European Hepatologic Collaboration (CEHC) on the Use of Thrombopoietin Receptor Agonists in Patients with Chronic Liver Disease Undergoing Invasive Procedures. J Clin Med 10 (22). <https://doi.org/10.3390/jcm10225419>

37. Matzdorff A, Meyer O, Ostermann H, Kiefel V, Eberl W, Kühne T, Pabinger I, Rummel M (2018) Immune Thrombocytopenia - Current Diagnostics and Therapy: Recommendations of a Joint Working Group of DGHO, ÖGHO, SGH, GPOH, and DGTI. Oncol Res Treat 41 Suppl 5:1-30. <https://doi.org/10.1159/000492187>

38. Neunert C, Terrell DR, Arnold DM, Buchanan G, Cines DB, Cooper N, Cuker A, Despotovic JM, George JN, Grace RF, Kühne T, Kuter DJ, Lim W, McCrae KR, Pruitt B, Shimanek H, Vesely SK (2019) American Society of Hematology 2019 guidelines for immune thrombocytopenia. Blood Adv 3 (23):3829-3866. <https://doi.org/10.1182/bloodadvances.2019000966>

39. Provan D, Arnold DM, Bussel JB, Chong BH, Cooper N, Gernsheimer T, Ghanima W, Godeau B, González-López TJ, Grainger J, Hou M, Kruse C, McDonald V, Michel M, Newland AC, Pavord S, Rodeghiero F, Scully M, Tomiyama Y, Wong RS, Zaja F, Kuter DJ (2019) Updated international consensus report on the investigation and management of primary immune thrombocytopenia. Blood Adv 3 (22):3780-3817. <https://doi.org/10.1182/bloodadvances.2019000812>

40. Sahu KK, Siddiqui AD, Rezaei N, Cerny J (2020) Challenges for management of immune thrombocytopenia during COVID-19 pandemic. Journal of medical virology 92 (11):2277-2282. <https://doi.org/10.1002/jmv.26251>
